# Supplementary material for: Long-Term Use and Application of Systematic Tailored Assessment for Responding to Suicidality (STARS) Protocol Following Original Training
Source: Int J Environ Res Public Health. 2022 Sep 8;19(18):11324. doi: 10.3390/ijerph191811324 (PMC9517291; doi:10.3390/ijerph191811324)
Supplement: Supplementary file 1 [file ijerph-19-11324-s001.zip › ijerph-1863290-supplementary.pdf]

Electronic Supplementary Material (ESM):

Table S1. Spearman Rho correlations between demographic and professional characteristics and use and perceptions of the STARS-p**.**

	All STARS	Part A	Part B	Part C	Safety plan	Clin Notes
Gender (Male vs female)	-.317**	-.264*	-.306*	-.360**	-.128	-.327**
Age	.074	.028	.249*	.004	.081	.256*
#Role (MHP-wQ vs MHP-woQ)	.233	-.161	-.156	.012	-.114	.050
Education (undergrad or less vs postgrad)	-.261*	-.023	-.006	-.138	-.075	-.040
Years of experience in the role that pertains to suicide prevention work	.091	.191	.104	.074	.196	.259*
FORMAL training in suicide prevention/management SINCE STARS training	.215	.109	.129	.151	.020	.260*
Frequency of INFORMAL training	-.180	-.111	.040	-.095	-.023	.011
Frequency of contact with suicidal persons as part of their role	.125	.033	-.075	.063	.083	.056
Loss of client by suicide	.080	-.112	-.191	.036	-.113	.063
Suicide attempt of client	.054	.274*	-.007	.209	.146	.120
Perception of ease of administration of the STARS-p	.573***	.275*	.320**	.308*	.552***	.497***
Perception of client feeling validated	.409***	.257*	.318**	.397***	.343**	.325**
Overall confidence in the usefulness of data	.422***	.259*	.222	.421***	.289*	.285*
Confidence in the use of STARS-p for informing needs-based priority areas or interventions to mitigate suicidality	.588***	.355**	.381**	.489***	.462***	.539***
Perception of STARS as a client-centred approach to determination of client's current suicidality	.517***	.178	.177	.342**	.537***	.366**

Note: N= 64-67; *$p \leq .05$, **$p \leq .01$, ***$p \leq .001$.

MHP-wQ = mental health practitioner with formal MH qualification; MHP-w0Q = mental health practitioner without formal MH qualification (e.g., trained peer or support workers).
